# A Bayesian Framework for Human Body Pose Tracking from Depth Image Sequences

**DOI:** 10.3390/s100505280

**Published:** 2010-05-25

**Authors:** Youding Zhu, Kikuo Fujimura

**Affiliations:** Honda Research Institute USA, 800 California Street, Mountain View, CA 94041-2810, USA; E-Mail: kfujimura@hra.com

**Keywords:** human pose tracking, depth images, Bayesian inference

## Abstract

This paper addresses the problem of accurate and robust tracking of 3D human body pose from depth image sequences. Recovering the large number of degrees of freedom in human body movements from a depth image sequence is challenging due to the need to resolve the depth ambiguity caused by self-occlusions and the difficulty to recover from tracking failure. Human body poses could be estimated through model fitting using dense correspondences between depth data and an articulated human model (local optimization method). Although it usually achieves a high accuracy due to dense correspondences, it may fail to recover from tracking failure. Alternately, human pose may be reconstructed by detecting and tracking human body anatomical landmarks (key-points) based on low-level depth image analysis. While this method (key-point based method) is robust and recovers from tracking failure, its pose estimation accuracy depends solely on image-based localization accuracy of key-points. To address these limitations, we present a flexible Bayesian framework for integrating pose estimation results obtained by methods based on key-points and local optimization. Experimental results are shown and performance comparison is presented to demonstrate the effectiveness of the proposed approach.

## 1. Introduction

For the past decades, human body pose tracking from video inputs has been an active research field motivated by various applications including human computer interaction, motion capture systems, and gesture recognition. The major challenges of recovering the large number of degrees of freedom in human body movements are the difficulties to resolve various ambiguities in the projection of human motion onto the image plane and the diversity of visual appearance caused by clothing and varying illumination.

**Figure 1.**
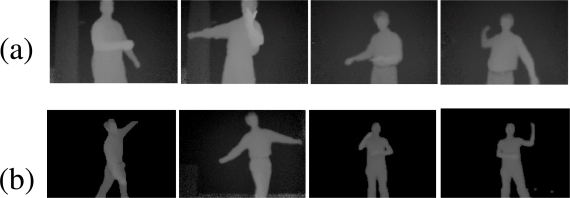
Depth data (a) Example upper body postures; (b) Example whole body postures.

Existing approaches for human pose tracking include methods based on single cameras, multiple cameras, and sensors beyond visible spectrum. Time-of-flight (TOF) based imaging devices have attracted researchers’ attention due to the potential to resolve depth ambiguity [1–4]. Robust pose tracking in 3D usually is difficult by using a single optical camera alone. In particular, methods based on silhouette information often fail to track 3D poses where there are self-occlusions. Although non-silhouette based methods [5, 6] have been proposed to track poses with self-occluded limbs, their robustness depends much on illumination conditions, body texture, and perhaps extensive training in case of learning based methods. Depth data, as in Figure 1, provides a valuable cue in resolving the depth ambiguity problem. Other advantages of TOF cameras include their portability, relatively good depth resolution compared with stereo cameras.

Most existing approaches to track human body pose from depth sequences [1–4] are related to the Iterative Closest Point (ICP) approach [7]. These approaches are able to track the human body pose with a high accuracy due to dense correspondences. However, these approaches based on local optimization are vulnerable to tracking failure when body parts get close to each other and often fail to recover from tracking failure afterwards. Knoop *et al.* [2] show that they can achieve more accurate pose tracking by integrating hand/face tracking. However, it becomes a challenging task to have a 2D hand/face tracker that works well for various complicated motion, and they do not elaborate on how the robustness of a 2D feature tracker could affect their 3D pose estimation. Zhu *et al.* [4] use coarse body identification to reduce the ambiguity during dense correspondence search. However, it has difficulties to detect arms when they re-appear.

Recovering from pose tracking failure is indeed an important component for a robust pose tracking algorithm. Considering example postures shown in Figure 1, on one hand, a visible arm could get so close to the torso that depth resolution is not high enough to detect the arm. Also, it is possible that a visible limb could be occluded temporarily by another limb. On the other hand, a missing limb can reappear later. A robust tracking algorithm must deal with intermittent occlusions to prevent tracking failures.

For many existing pose tracking methods, tracking long sequences will result in tracking failure which cannot be easily recovered. This paper presents a key-point based method to reconstruct poses from anatomical landmarks detected and tracked from depth image analysis. The key-point based method is robust and can recover from tracking failure when a body part is re-detected and tracked. However, its pose estimation accuracy depends solely on the image-based localization accuracy of key-points. To address these limitations, we present a Bayesian framework to integrate pose estimation results from methods using local optimization and key-point detection. Our contribution of the work is to integrate pose estimation results from multiple methods. In particular, we use results obtained by using key-points and local optimization and show that accuracy is improved compared with either method alone.

The rest of the paper is organized as follows. Section 2 introduces the human model used in this paper, and the background on pose estimation with constrained inverse kinematics. Our Bayesian method for accurate and robust pose tracking is presented in Section 3. Methods using key-points and local optimization are described in Subsections 3.1 and 3.2, respectively. Experimental results are shown in Section 4. Section 5 concludes the paper.

## 2. Human Body Model and Pose Estimation with Constraint Inverse Kinematics

**Figure 2.**
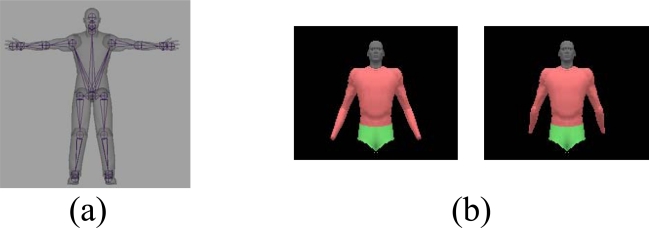
Human body model (a) Hierarchical joint link model with 28 dofs; (b) Elbow joint limit constraints for natural pose tracking.

The human body model is represented as a hierarchy of joint link models with a skin mesh attached to it as in Lewis *et al.* [8]. The human model in Figure 2(a) includes 28 dofs for whole body, and 20 dofs for upper body. During pose estimation, one natural constraint is to enforce joint limits. For example, by enforcing elbow joint limits, we could avoid generating the backward bending arms as in the Figure 2(b).

Let *q*_0_ be the initial model pose, *V* be the set of model marker points, and *P* be the set of observed points from the sensor. Let *q̂* = *ConstraintIK*(*q*_0_*, V, P*) denote the constrained inverse kinematics as:

(1) $$\hat{q}=q_{0}+sJ^{*}\left(P-V\right)$$

(2) $$J^{*}=W_{1}^{-1}J^{T}{\left(JW_{1}^{-1}J^{T}+W_{2}\right)}^{-1}$$

(3) $$J={\left[\begin{array}{ccccc} J_{1}^{T} & \cdots & J_{i}^{T} & \cdots & J_{N}^{T} \end{array}\right]}^{T}$$

where *J* is the augmented Jacobian matrix, *J_i_* is Jacobian for *i*th model vertex, *s* is a scalar to adjust the step size of inverse kinematics, *W*_1_ and *W*_2_ are defined for singularity avoidance and joint limit avoidance. This type of formulation using inverse kinematics is often used to derive manipulators orientation at each joint, when given a desired position of the end-effector. See Zhu *et al.* [9] for more details.

**Figure 3.**
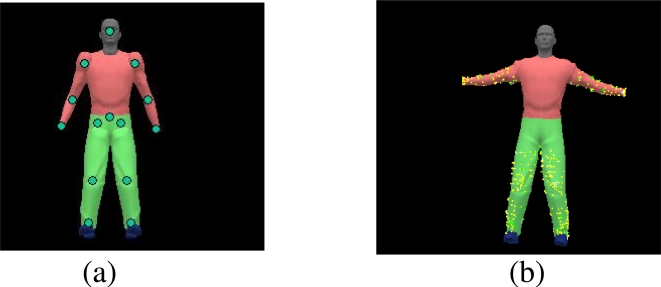
Model marker points (a) from key-point detection; (b) from dense ICP correspondences (each yellow vector represents a correspondence pair).

Our model marker points (for key-point detection) include the set of model vertices as shown in Figure 3. In Figure 3(a), model marker points are located at the human anatomical landmarks, and observed points are detected through low-level depth image analysis as described in Subsection 3.1. On the contrary, for model fitting, model marker points are sampled randomly from the model vertices (Figure 3(b)), and observed points are found during the ICP correspondence search as described in Subsection 3.2.

## 3. Robust 3D Pose Tracking with Bayesian Method

**Figure 4.**
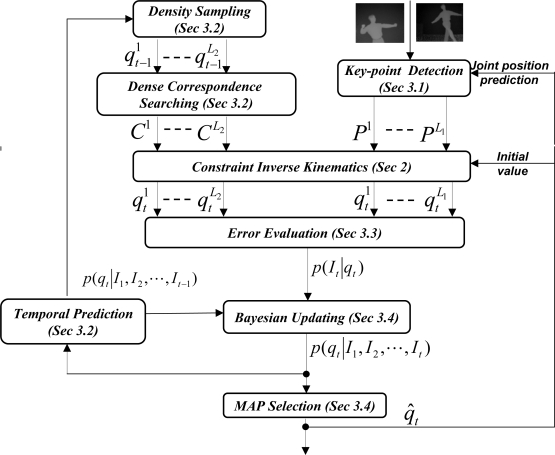
Robust pose estimation with Bayesian tracking framework.

The main idea of tracking is illustrated in Figure 4. Two contrasting methods are used independently to give estimates of a human pose. The righthand side of Figure 4 represents a sparse method (based on tracking several anatomical features of the body), while the left-hand side of Figure 4 represents a dense method (tracking based on a mesh representation of the body). Each method generates hypothesis of the current pose (where output formats are a mesh and a set of anatomical landmark points, respectively) using the time sequence obtained so far. The results are integrated to produce the best estimate.

Let *q_t_* be the model pose parameters, including all degrees of freedom of the human model at time *t*, and *p*(*q_t_|I*_1_*, I*_2_, · · · *, I_t_*) be the probability distribution of pose parameters given all observed images { *I*_1_*, I*_2_*, · · ·, I_t_*}, then Bayesian tracking is formulated as:

(4) $$\begin{array}{l} p\left(q_{t}|I_{1},I_{2},\cdots ,I_{t}\right)\propto p\left(I_{t}|q_{t}\right)p\left(q_{t}|I_{1},I_{2},\cdots ,I_{t-1}\right)\\ \;\;\;\;\;\;\;\;\;\;\;\;\;\;=p\left(I_{t}|q_{t}\right)\int_{q_{t-1}}p\left(q_{t}|q_{t-1}\right)p\left(q_{t-1}|I_{1},I_{2},\cdots ,I_{t-1}\right)dq_{t-1} \end{array}$$

Let us assume that we can approximate the observation distribution as mixture of Gaussian:

(5) $$p(I_{t}|q_{t})=\sum_{k=1}^{K}w_{k}^{t}N\left(q_{t};\mu_{k}^{t},\Lambda_{k}^{t}\right)$$

where $N(q_{t};\mu_{k}^{t},\Lambda_{k}^{t})$ denotes that *q_t_* has a Gaussian distribution with mean *q_t_* and covariance $\Lambda_{k}^{t}$.

Let human dynamics have Gaussian noise *N*(0, *W*), the temporal propagation is given by:

(6) $$p\left(q_{t}|I_{1},I_{2},\cdots ,I_{t-1}\right)=\sum_{j=1}^{M}\pi_{j}^{t-1}N\left(q_{t};f\left(\mu_{j}^{t-1}\right),\Lambda_{j}^{t-1}+W\right)$$

where $f\left(\mu_{j}^{t-1}\right)$ is any appropriate pose dynamic process and *π*’s are weights.

Using the above Bayesian tracking equation, we can represent the posterior probability distribution as:

(7) $$p\left(q_{t}|I_{1},I_{2},\cdots ,I_{t}\right)=\sum_{k=1}^{K}w_{k}^{t}N\left(q_{t};\mu_{k}^{t},\Lambda_{k}^{t}\right)\sum_{j=1}^{M}\pi_{j}^{t-1}N\left(q_{t};f\left(\mu_{j}^{t-1}\right),\Lambda_{j}^{t-1}+W\right)$$

which will be, in general, a mixture of *K* × *M* Gaussian components. As we can see, this will result in an exponential increase of Gaussian components for the posterior probability distribution along the updating of time. To prevent this exponential increase in Gaussian components, we approximate it with *M* component Gaussian-mixture distribution:

(8) $$p\left(q_{t}|I_{1},I_{2},\cdots ,I_{t}\right)\approx \sum_{j=1}^{M}\pi_{j}^{t}N\left(q_{t};{\hat{\mu }}_{j}^{t},{\hat{\Lambda }}_{j}^{t}\right)$$

Such an approximation is reasonable in our pose estimation method as we integrate the data-driven estimation as described in Subsections 3.1. and 3.2. into Bayesian update as described in Subsection 3.4., and these data-driven estimation is very effective to allow us only maintaining a small number of pose hypotheses during the tracking.

Since we represent the posterior probability distribution as a sum of Gaussian, there are available methods to perform density approximation. One simple way is to keep the dominant modes in the posterior probability distribution. Researchers [5, 10] also suggest to pick modes from a likelihood function and combine them with compatible ones from the predicted prior probabilities. Some authors [11] also pick the modes from a likelihood function and re-weight with predicted prior probability.

The detailed illustration of this Bayesian inference method to pose tracking is shown in Figure 4, where we are able to integrate three sources of information: key-point detection from low-level image analysis, local pose optimization with ICP, and temporal prediction information if that is available. We describe these components in the following subsections.

### 3.1. Key-Point Detection from Depth Image Sequence for Pose Tracking

#### 3.1.1. Body part detection

**Figure 5.**
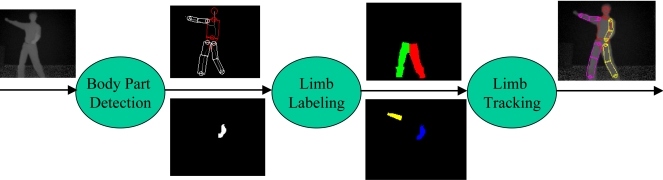
Body part detection, labeling and tracking.

**Figure 6.**
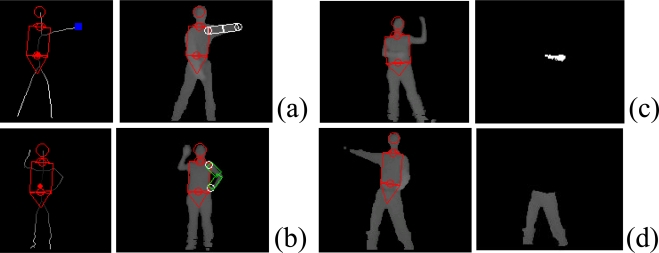
HNT template localization (shown in red) and limb detection: (a) Open arm detection; (b) Looped arm detection; (c) Arm detection that is in front of torso; (d) Lower limb detection.

In order to have a robust pose tracker, one of the crucial processing steps is to localize each visible limb. We present a method to detect, label and track body parts using depth images as shown in Figure 5. To detect major body parts such as the head, torso, and waist, we make use of a deformable template referred to as the HNT template which consists of a head, neck, and trunk. The trunk is further decomposed into a torso and waist. They are represented by a circle, trapezoid, rectangle, and another trapezoid, respectively as in Figures 5 and 6. To localize the HNT template, our algorithm takes a background-subtracted depth image *I* as input and deforms the HNT template to produce the optimal template configuration by minimizing the discrepancy between the deformed HNT template and the background-subtracted depth image. See Zhu *et al.* [9] for more details about HNT template and its detection algorithm.

Once the head, neck, and trunk are detected, limbs (two arms and two legs) are to be detected as shown in Figure 6. For example, we can detect a upper body limb that is open, or that forms a loop, or that is in front of torso based on depth image analysis. We can detect lower limbs by finding all pixels that are lower than the waist.

#### 3.1.2. Labeling

After the limbs are detected, we perform a labeling step in order to differentiate the left and right limbs as well as to determine the limb occlusion status. We use the following steps to label detected arms (same steps applied to leg labeling) based on the arm occlusion status at the last frame. For image frames where both arms are visible (in previous frame), let us define *H*_LA_ and *H*_RA_ to be the histograms of depth values for the left and right arms respectively, and we assign each pixel *x* in detected limb a label *L_x_* (either Left or Right) based on its geometric and appearance distance to the tracked arms. Likelihood of *x* being Left Arm (LA) or Right Arm (RA) is computed by using the following formula:

(9) $$P\left(L_{x}^{t}=\text{LA}|X_{\text{LA}}^{t},H_{\text{LA}}^{t},X_{\text{RA}}^{t},H_{\text{RA}}^{t}\right)=\frac{e^{-\gamma d_{\text{LA}}\left(x\right)}H_{\text{LA}}\left(I_{x}\right)}{e^{-\gamma d_{\text{LA}}\left(x\right)}H_{\text{LA}}\left(I_{x}\right)+e^{-\gamma d_{\text{RA}}\left(x\right)}H_{\text{RA}}\left(I_{x}\right)}$$

where *X^t^* represents configuration at time *t* and *d*_LA_(*x*) is the distance from pixel *x* to the left arm:

(10) $$d_{\text{LA}\left(x\right)}=\{\begin{array}{ll} 0 & \text{if }x\;\text{is}\;\text{inside}\;\text{left}\;\text{arm}\\ d\left(x,\text{LA}\right) & \text{otherwise} \end{array}$$

where *d*(*x,* LA) is the minimal distance from *x* to edges of the left arm. *d*_RA_(*x*) is defined similarly. In short, a pixel *x* has a high probability of belonging to LA, if *x* is sufficiently close to where LA was in the previous frame. While two arms are overlapping in the image, *x* has a high probability of belonging to LA if it has a depth value that is close to depth values represented by the left arm in the previous frame.

When only one arm is visible from the last frame, we compute the geometric distance from the detected arm pixels to the tracked arm, and decide the label based on the maximal arm movement distance between successive frames. When both arms are not visible from the last frame, we label the detected arm based on its spatial distribution relative to the torso center line, where the left arm is located to the left of torso center line.

Finally, when the observed number of pixels for a limb is less than the threshold, we declare that the limb is occluded. For each visible limb, we preform a local optimization to align the 2-D scaled prismatic model [12] to the detected limbs.

#### 3.1.3. Pose hypotheses from features

Key-points corresponding to the human anatomical landmarks as in Figure 3(a) are extracted from the deformed HNT template and the aligned 2-D scaled prismatic model. Due to self-occlusions, we might only be able to detect a subset of landmarks at any frame. In our Bayesian framework, we use these bottom-up depth image analysis results to improve the robustness of pose estimation and recover from tracking failure.

Referring to Figure 4, let *Ps* denote the extracted Key-points, and let *Ms* denote the corresponding subset of human anatomical landmarks. We then generate 3D pose hypotheses based on constrained inverse kinematics (defined at Equation 1). Without loss of generality, let us denote it as:

(11) $$\hat{q}=\mathrm{Constraint}\;I\;K\;\left(q_{0},Ms,Ps\right)$$

For certain poses (e.g., straight arm), we can only obtain approximate elbow positions. Also, the estimated pose based on constrained inverse kinematics depends on starting pose values *q*_0_. Let *q̂*_*t*–1_ be the optimal pose estimation from the last frame and let $q_{t-1}^{0}$ be the human resting pose. We use the constrained inverse kinematics to generate three sets of pose hypotheses (*L*_1_ = 3 as in Figure 4):

H1: $q_{t}^{1}$ is the pose generated based on both the optimal estimation *q̂*_*t*–1_ and all feature points.
H2: $q_{t}^{2}$ is the pose generated based on the resting pose $q_{t-1}^{0}$ and all feature points. This hypothesis is useful to prevent the possibly erroneous estimation from the last frame.
H3: $q_{t}^{3}$ is the pose generated based the optimal estimation *q̂*_*t*–1_ without using the extracted elbow feature points. This hypothesis is useful to prevent the large error in elbow detection and extraction.

### 3.2. Temporal Prediction, Density Sampling and Dense Correspondence Searching for Pose Tracking

Since the motion to be tracked in this study is general and has high uncertainty, a common approach is to model the human pose temporal dynamics as zero velocity with a Gaussian noise *N*(0, *W*). Therefore, we can approximate the temporal prediction prior probability distribution as:

(12) $$p\left(q_{t}|I_{1},I_{2},\cdots ,I_{t-1}\right)=\sum_{j=1}^{M}\pi_{j}^{t-1}N\left(q_{t};\mu_{j}^{t-1},\Lambda_{j}^{t-1}+W\right)$$

Density sampling can be performed based on this temporal prediction prior probability distribution as this is a standard Gaussian mixture distribution.

Let $q_{t-1}^{i}$ be one of samples from density sampling, *V s* denote a set of sampled model vertices that is visible from camera, *C s* denote the set of 3D depth points that is closest to *V s* (as shown in Figure 3(b)), and $q_{t}^{i}$ denote the pose from local pose optimization:

(13) $$q_{t}^{i}=\mathrm{Constraint}\;I\;K\;\left(q_{t-1}^{i},Vs,Cs\right)$$

We obtain visible model vertices *V s* from the depth buffer technique of OpenGL rendering. Closest point set *C s* is obtained through its grid acceleration data structure.

### 3.3. Tracking Error Evaluation

To evaluate tracking quality, we use a tracking error measurement function that is based on the sum of the distances from sampled depth points to their corresponding closest model vertices. Without loss of generality, let us use *P s* to denote the set of sampled depth points and *V s* the set of visible model vertices that are closest to the *P s*. Then, our tracking error measurement function is defined as:

(14) $$d^{2}\left(Ps,Vs\left(q_{t}\right)\right)=\sum_{j}{\left\|Ps_{j}-Vs_{j}\left(q_{t}\right)\right\|}^{2}$$

With this tracking error measurement function, we can approximate the observation distribution as:

(15) $$p\left(I_{t}|q_{t}\right)\propto \exp\left\{-d^{2}\left(Ps,Vs\left(q_{t}\right)\right)\right\}$$

We can further approximate the observation distribution by keeping only a few modes from the local optimization and constrained inverse kinematics on key-points. Let { $\mu_{k}^{t}$, *k* = 1, · · ·, *k* = *K*} denote the set of modes, we can approximate the observation distribution as:

(16) $$p\left(I_{t}|q_{t}\right)\approx \sum_{k=1}^{K}w_{k}^{t}N\left(q_{t};\mu_{k}^{t},\Lambda_{k}^{t}\right)$$

where, $w_{k}^{t}$ can be estimated as:

$${\tilde{w}}_{k}^{t}\approx \exp\left\{-d^{2}\left(Ps,Vs\left(\mu_{k}^{t}\right)\right)\right\}$$

(17) $$w_{k}^{t}=\frac{{\tilde{w}}_{k}^{t}}{\sum_{k=1}^{K}{\tilde{w}}_{k}^{t}}$$

$\Lambda_{k}^{t}$ can be estimated as:

(18) $$\Lambda_{k}^{t}\approx {\left(J_{Vs}^{T}J_{Vs}\right)}^{-1}$$

### 3.4. Bayesian Updating and MAP Selection

Given observation distribution *p*(*I_t_|q_t_*) as Equation 16, and temporal prediction prior probability distribution *p*(*q_t_|I*_1_, *I*_2_, · · ·, *I*_*t*–1_) as Equation 12, we obtain the posterior probability distribution as:

(19) $$p\left(q_{t}|I_{1},I_{2},\cdots ,I_{t}\right)=\sum_{k=1}^{k}w_{k}^{t}N\left(q_{t};\mu_{k}^{t},\Lambda_{k}^{t}\right)\sum_{j=1}^{M}\pi_{j}^{t-1}N\left(q_{t};\mu_{j}^{t-1},\Lambda_{j}^{t-1}+W\right)$$

In order to avoid the exponential increase of Gaussian components, without loss of generality, we first approximate it by the first *M* dominant observation modes as:

(20) $$p\left(q_{t}|I_{1},I_{2},\cdots ,I_{t}\right)\approx \sum_{k=1}^{M}{\hat{w}}_{k}^{t}N\left(q_{t};\mu_{k}^{t},\Lambda_{k}^{t}\right)\sum_{j=1}^{M}\pi_{j}^{t-1}N\left(q_{t};\mu_{j}^{t-1},\Lambda_{j}^{t-1}+W\right)$$

and then re-weight them with temporal prior probability:

(21) $$p\left(q_{t}|I_{1},I_{2},\cdots I_{t}\right)\approx \sum_{j=1}^{M}\pi_{j}^{t}N\left(q_{t};\mu_{j}^{t},\Lambda_{j}^{t}\right)$$

where weights $\pi_{j}^{t}$ can be estimated as:

(22) $${\tilde{w}}_{j}^{t}={\hat{w}}_{j}^{t}\sum_{k=1}^{M}\pi_{k}^{t-1}N\left(\mu_{j}^{t};\mu_{k}^{t-1},\Lambda_{k}^{t-1}+W\right)$$

(23) $$\pi_{j}^{t}=\frac{{\tilde{w}}_{j}^{t}}{\sum_{j=1}^{M}{\tilde{w}}_{j}^{t}}$$

At any frame, the optimal pose estimation is exported as the mode in the posterior probability distribution *p*(*q_t_|I*_1_*, I*_2_*, · · ·, I_t_*).

## 4. Experiments

The Bayesian pose tracking algorithm is implemented and tested on a set of upper and whole body sequences captured from a single time-of-flight (TOF) range sensor [13] at 16 frame per second. Upper body data sequences are captured with a distance between 1.5 m and 2 m, and whole body data sequences are captured with a distance around 3 m. Each sequence has a duration about between 10 to 15 s. Through experiments, our major goal is to show that

- The proposed Bayesian framework is able to track robustly and recover from tracking failure by integrating low-level key-point detection from depth image analysis;
- The proposed Bayesian framework is able to achieve a higher accuracy by taking advantage of the ICP to refine the alignment between 3D model and point clouds;

To that end, the captured data sequences include the type of human motion where it has self-occlusions between body parts. As a result, these captured data sequences are complicated, and previous methods based on local optimization fail to track them because of self-occlusion. Local optimization methods have especially poor performance to track such scenario where limb disappears and reappears again during the motion.

**Figure 7.**
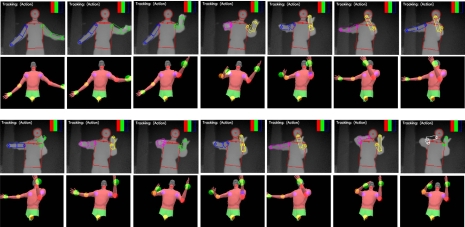
Upper body pose tracking for violin playing motion. Rows 1 and 3: depth image sequence with the detected body parts. Rows 2 and 4: corresponding reconstructed pose.

**Figure 8.**
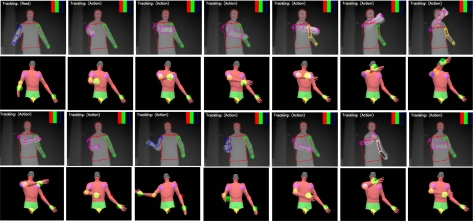
Upper body pose tracking for frisbee throwing motion. Rows 1 and 3: depth image sequence with the detected body parts. Rows 2 and 4: corresponding reconstructed pose.

**Figure 9.**
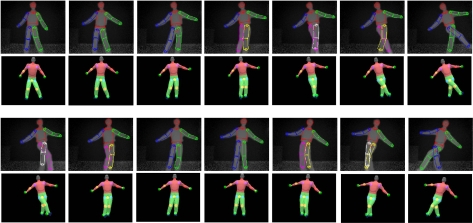
Whole body pose tracking with self occlusions during leg crossing. Rows 1 and 3: depth image sequence with the detected body parts. Rows 2 and 4: corresponding reconstructed pose.

**Figure 10.**
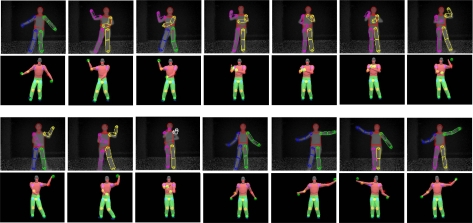
Whole body pose tracking during a dancing sequence. Rows 1 and 3: depth image sequence with the detected body parts. Rows 2 and 4: corresponding reconstructed pose.

Our current implementation works well for body twists up to 40 degree rotation on either side of a front facing posture. Large twists and severe interaction between upper and lower body limbs remain as a challenge in the current implementation. Example upper-body and whole-body tracking results are shown in Figures 7–10. In all of our experiments, we use *K* = *L*_1_ + *L*_2_ = 3 + 3 = 6, *M* = 3 (refer to Equation 7). Currently these values are selected based on the empirical method. Firstly, we select *L*_1_ = 3 as explained in Subsection 3.1. Secondly, *L*_2_ = 3 and *M* = 3 are empirically selected based on the performance of the tracker on the example motion sequences in our database. Increasing *L*_2_ and *M* could improve accuracy further, but could also slow down the tracking dramatically. In contrast with our current brute-force implementation, we are seeking other implementation methods such as parallel programming techniques to take advantage of inherent parallelism between the hypothesis computations.

We summarize and compare its performance with the ICP method and key-point based method as in Table 1. The ICP method utilizes general correspondences to estimate the pose, which does not require tracking of key-points. Nevertheless, the ICP method could result in tracking failure for transient occlusions, and is difficult to recover from it. Furthermore, the ICP method could not be integrated with other information flexibly. The key-point based method is able to track through transient occlusion, and recover from tracking failures when the body parts are detected again. However, it is not able to take advantage of other information. As seen, the Bayesian-based framework is able to take advantage of both ICP and key-point based methods. It is able to track through transient occlusions, recover from tracking failure whenever body parts are detected again, and update the pose by performing local optimization without key-points. The Bayesian-based framework has the potential to make use of other information flexibly whenever available, for example, pose prediction from machine learning approaches. Furthermore, the Bayesian-based framework could achieve a higher accuracy for joint trajectories than key-point based methods because it could take advantage of ICP to refine the alignment between 3D model and point clouds, as shown in Table 2.

## 5. Conclusions and Future work

We have presented a Bayesian framework for human pose tracking from depth image sequences. Human pose tracking remains as a challenging problem, primarily because pose is difficult to track due to occlusion, fast movements, and ambiguity. Generating multiple hypotheses for human pose for one image is at times necessary to arrive at a correct solution. A method has been proposed to demonstrate a potential to integrate pose estimation results from different modalities to improve the robustness and accuracy. We believe the parallel nature of the hypothesis evaluation permits us to achieve a faster implementation with latest parallel programming techniques.

## Figures and Tables

**Table 1.** Comparison between various human pose tracking approaches.

Methods	Tracking through occlusion	Error-recovery	Tracking with missing key-points	Integration with other information	Speed
ICP based method	No	No	Yes	No	5∼9 Hz
Key-point based method	Yes	Yes	No	No	3∼6 Hz
Bayesian-based method	Yes	Yes	Yes	Yes	0.1 Hz

**Table 2.** A comparison of overall trajectory accuracy between key-point based method and Bayesian-based method.

Methods	X trajectory accuracy	Y trajectory accuracy	Z trajectory accuracy
Key-point based method	80 mm	84 mm	93 mm
Bayesian-based method	73 mm	78 mm	87 mm
